# 

**Published:** 1902-10

**Authors:** 


### BUFFALO MEDICAL JOURNAL

**Established 1845 by AUSTIN FLINT, M. D.**

EDITOR:

WILLIAM WARREN POTTER, M. D.

ASSISTANT EDITORS:

WILLIAM C. KRAUSS, M. D. NELSON W. WILSON, M. D.

ASSOCIATE EDITORS:

JAMES WRIGHT PUTNAM, M. D.
ERNEST WENDE, M. D.
JOHN PARMENTER, M. D.
JOHN A. MILLER, PH. D.
HARVEY R. GAYLORD, M. D.
MAUD J. FRYE, M. D.

SCRIPTA FERUNT ANNOS

LIBRARY
SURGEON GENERAL'S OFFI[illegible]
6 - [illegible] - 1902

EUROPEAN AGENCY, J. B. BAILLIÈRE & FILS, 19 Rue Hautefeuille, Paris.

*Subscription, if paid in advance, $2.00; otherwise, $2.50. Foreign subscription, $2.50*

Entered at the Post Office at Buffalo, N. Y., as second-class mail matter.

A. T. BROWN PRINTING HOUSE, BUFFALO, N. Y.

Table of Contents on Page V.

